# Vascular Malformation in the Gastrointestinal Tract Mimicking Vasculitis: A Case Report

**DOI:** 10.7759/cureus.79814

**Published:** 2025-02-28

**Authors:** Luiz Felipe Tojal Ramos dos Santos, Camila Gusmão Vicente de Carvalho, André Luíz Gioia Morrell, André Silva Franco

**Affiliations:** 1 Rheumatology, Hospital das Clínicas da Faculdade de Medicina da Universidade de São Paulo (FMUSP), São Paulo, BRA; 2 Gastroenterology, Instituto Morrell, São Paulo, BRA

**Keywords:** anca-associated vasculitis, eosinophilic granulomatosis with polyangiitis (egpa), gastrointestinal hemangioma, lower gastrointestinal hemorrhage, vasculitis mimickers

## Abstract

Diagnosing vasculitis is challenging because it lacks pathognomonic signs and symptoms. Gastrointestinal vasculitis further complicates the picture, given its high mortality risk and the potential absence of systemic manifestations; thus, a systematic approach that includes ruling out vasculitis mimickers is useful. We report the case of a 29-year-old male evaluated for suspected intestinal vasculitis due to recurrent rectal bleeding, weight loss, fatigue, elevated inflammatory markers, high immunoglobulin E (IgE) levels, and positive anti-neutrophil cytoplasmic antibodies (ANCA), coupled with computed tomography (CT) showing diffuse parietal thickening of the anus, rectum, and sigmoid colon. These findings raised suspicion for eosinophilic granulomatosis with polyangiitis, prompting high-dose corticosteroid therapy before definitive diagnosis. However, contrast-enhanced magnetic resonance imaging (MRI) later revealed phleboliths and venous lakes, indicating gastrointestinal diffuse cavernous hemangioma (GDCH). Sirolimus was introduced to facilitate steroid tapering and to reduce bleeding, ultimately leading to significant lesion regression and symptom resolution. This case underscores the importance of excluding vasculitis mimickers - particularly vascular malformations - when evaluating potential gastrointestinal vasculitis.

## Introduction

Vasculitis is a group of systemic inflammatory diseases characterized by the involvement of blood vessel walls, classified based on the size and type of affected vessels [1]. Gastrointestinal involvement varies widely depending on the underlying vasculitis subtype, with clinical manifestations ranging from mild symptoms to life-threatening complications.

Large-vessel vasculitis may result in widespread intestinal infarction, in contrast to small-vessel vasculitis, which presents with segmental ischemia, ulcerations, and bleeding [2]. Among small-vessel vasculitis, the most frequently associated with gastrointestinal manifestations are immunoglobulin A (IgA) vasculitis, polyarteritis nodosa, and anti-neutrophil cytoplasmic antibody (ANCA)-associated vasculitis, particularly eosinophilic granulomatosis with polyangiitis [3].

Patients with gastrointestinal vasculitis can exhibit abdominal pain, nausea, vomiting, diarrhea, or hematochezia, either in isolation or alongside systemic manifestations. Given the high risk of hemorrhage associated with intestinal biopsy, the diagnosis of gastrointestinal vasculitis generally hinges on clinical and laboratory findings. As a result, a thorough investigation to exclude vasculitis mimickers is essential.

Gastrointestinal diffuse cavernous hemangioma (GDCH) is a rare vascular malformation that can mimic intestinal vasculitis. Most patients present with painless intraluminal bleeding and chronic iron-deficiency anemia. Due to its rarity, misdiagnosis is common, leading to inappropriate surgical interventions in up to 80% of cases [4]. Magnetic resonance imaging (MRI) remains pivotal for GDCH diagnosis because of its characteristic imaging findings, such as phleboliths or venous lakes.

Herein, we report a case of GDCH in a patient with high immunoglobulin E (IgE) levels and positive ANCA, highlighting its potential as a vasculitis mimicker and emphasizing the importance of distinguishing between these entities to guide appropriate management.

## Case presentation

A 29-year-old male was referred to a rheumatology clinic for evaluation of suspected gastrointestinal vasculitis after experiencing recurrent rectal bleeding for four weeks, accompanied by weight loss and fatigue. His medical history included sporadic episodes of hematochezia since the age of 20, previously attributed to hemorrhoids. Otherwise, he was in good health.

A prior workup by a gastrointestinal surgeon revealed a significant drop in hemoglobin levels (from 16.0 g/dL to 11 g/dL over the past month), elevated inflammatory markers, positive ANCA in an atypical pattern (1/320), and high IgE levels (Table 1).

**Table 1:** Laboratory findings ANCA anti-neutrophil cytoplasmic antibodies; P-ANCA: perinuclear anti-neutrophil cytoplasmic antibodies; Ig: immunoglobulin.

Laboratory Test	Result	Reference Range
Hemoglobin	11.0 g/dL	13.3-16.5 g/dL
Hematocrit	33.8%	39.2-49.0%
Leukocytes count	5,410/mm³	3,650-8,120/mm³
Platelet count	377,000/mm³	151,000-304,000/mm³
Ferritin	239 µg/L	26-446 µg/L (Male)
Iron	44 µg/dL	65-175 µg/dL
Total iron binding capacity	267 µg/dL	250-425 µg/dL
Transferrin saturation	17%	20-50%
ANCA	1/320 (atypical p-ANCA)	Negative
Anti-nuclear antibodies	Negative	Negative
IgM	391 mg/dL	50-300 mg/dL (≥18 years)
IgG	1603 mg/dL	600-1500 mg/dL (≥18 years)
IgA	694 mg/dL	50-400 mg/dL (≥18 years)
IgE	1321 kU/L	<100 kU/L (>16 years)
Erythrocyte sedimentation rate	89 mm	2-28 mm (Male, 1st hour)

**Figure 1:**
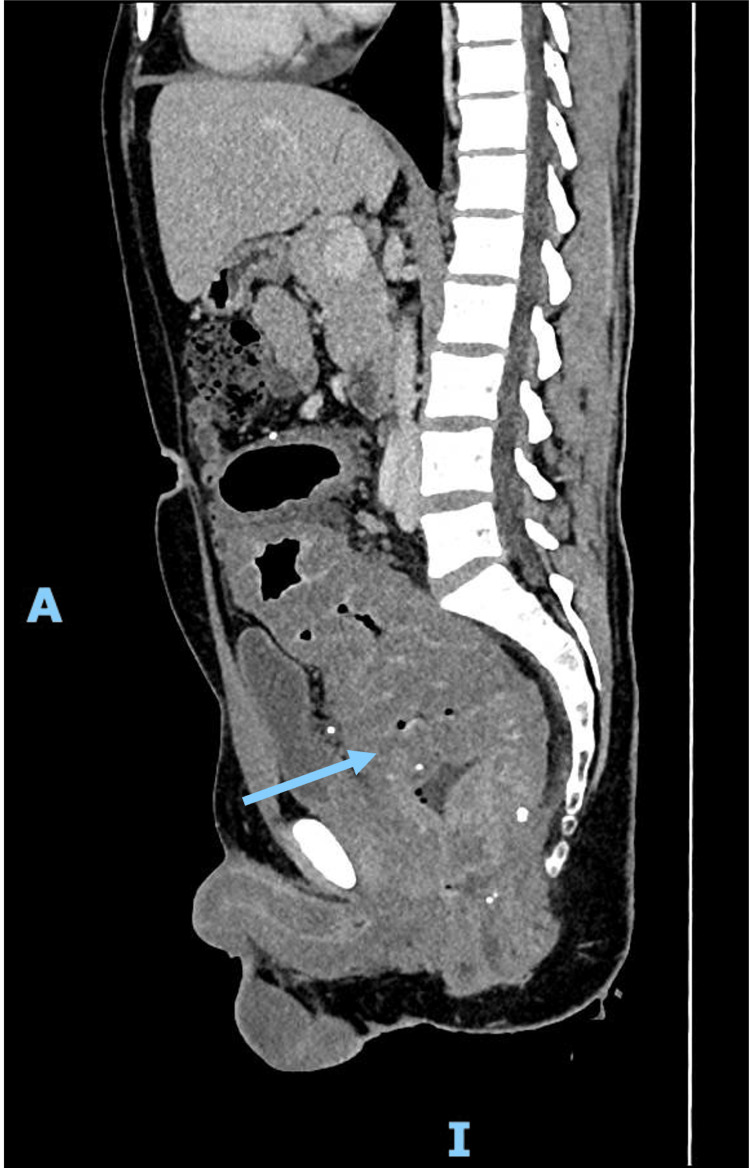
CT scan of the abdomen and pelvis Marked diffuse parietal thickening of the anal canal, rectum, and sigmoid colon, extending up to the transition with the left colon over approximately 50 cm. The findings are more prominent in the rectum and anal canal, where there is apparent luminal narrowing.

Computed tomography (CT) demonstrated diffuse parietal thickening extending approximately 50 cm along the anus, rectum, and sigmoid colon (Figure 1). Given these findings, eosinophilic granulomatosis with polyangiitis was suspected, prompting his referral for further assessment.

**Figure 2:**
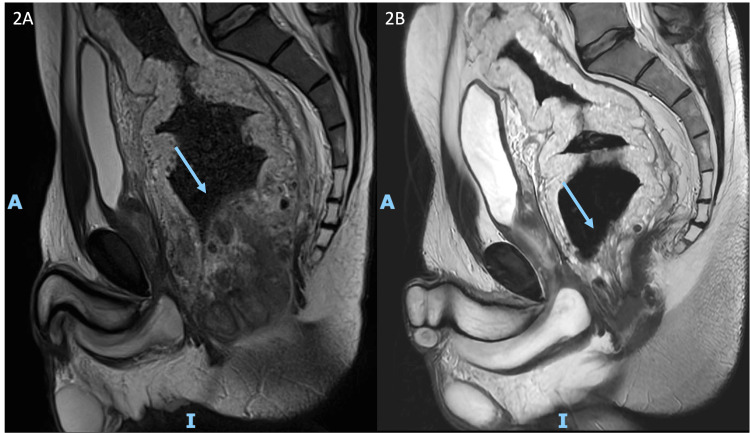
Contrast-enhanced MRI of the pelvis (a) MRI showing venous lakes in the rectum and anus (arrow). (b) MRI after six months with significant reduction of venous lakes

Due to the severity of symptoms and the potential for a life-threatening condition, empiric treatment with methylprednisolone pulse therapy (500 mg for three days) was initiated before a definitive diagnosis was established. Two weeks post-treatment, rectal bleeding had completely resolved, and laboratory tests showed normalization of hemoglobin levels and inflammatory markers. However, contrast-enhanced MRI confirmed persistent parietal thickening and further identified phleboliths and venous lakes in the rectum and anus, suggestive of GDCH (Figure 2A).

Surgical resection was deemed unsuitable due to the high risk of sphincter dysfunction and the potential need for permanent colostomy. To facilitate corticosteroid tapering and prevent relapse, oral sirolimus (2 mg/day) was initiated. The patient remained asymptomatic, with no recurrence of rectal bleeding, and follow-up MRI after six months demonstrated significant regression of venous lakes (Figure 2B). He regained weight and showed sustained clinical improvement following sirolimus therapy.

## Discussion

We report a case of GDCH with high IgE levels and positive ANCA, initially raising suspicion for ANCA-associated vasculitis, particularly eosinophilic granulomatosis with polyangiitis. Glucocorticoid therapy was promptly initiated based on this suspicion. However, the absence of systemic involvement and a specific ANCA pattern suggested an atypical presentation of vasculitis, making it essential to rule out vasculitis mimickers before establishing a definitive diagnosis.

Gastrointestinal vasculitis presents with a highly variable clinical spectrum, as different parts of the digestive tract can be affected, though the small intestine and large bowel are most commonly involved [2]. Mesenteric vessel inflammation, microaneurysms in medium-sized vessels, and inflammatory involvement of the liver and pancreas can all lead to ischemic lesions [3]. The presence of gastrointestinal vasculitis is a prognostic marker, as it carries a high risk of bleeding and perforation, necessitating urgent intervention [5].

Isolated gastrointestinal vasculitis is classified as single-organ vasculitis, often manifesting as abdominal pain, nausea, diarrhea, hematochezia, or melena, and may precede systemic vasculitis. Localized vasculitis of the intestinal tract is associated with significant morbidity and mortality [3,6]. Given the potential severity of this condition, differentiating true gastrointestinal vasculitis from its mimickers is crucial to avoid misdiagnosis and inappropriate treatment.

Several diseases can mimic gastrointestinal vasculitis, particularly conditions causing mesenteric ischemia. These include atherosclerosis, thromboembolic conditions, antiphospholipid syndrome, superior mesenteric artery syndrome, and thromboangiitis obliterans [2]. Additionally, complications of immunosuppressive therapy in patients with known vasculitis, such as glucocorticoid-induced ulcerations, gastrointestinal bleeding, and visceral perforations, can be challenging to distinguish from disease flares [7]. Moreover, immunosuppressed patients are at increased risk of gastrointestinal infections, which can further complicate the differential diagnosis [2].

GDCH is a rare vascular malformation of the gastrointestinal tract that can closely mimic vasculitis [4]. Patients often present with chronic abdominal pain, recurrent infections, nonspecific gastrointestinal bleeding, clotting disorders, functional impairments, and physical deformities [8]. Due to its overlapping clinical features with vasculitis, radiological studies play a crucial role in distinguishing GDCH from inflammatory conditions.

On MRI, transmural bowel-wall thickening with or without phleboliths is considered pathognomonic for GDCH [9]. Ultrasound and angiography can further assist in diagnosis [9]. However, biopsy should be strictly avoided due to the high risk of hemorrhage [10]. Accurate diagnosis is essential to ensure proper management and prevent unnecessary interventions.

Several treatment options exist for GDCH. Medical therapy includes glucocorticoids and sirolimus, which has proven effective in reducing pain and deformity associated with the condition [8,11]. Surgical management focuses on sphincter-preserving procedures, such as low anterior resection with minimal rectal cuff preservation, rectal mucosectomy, and coloanal pull-through techniques [12].

This case highlights the importance of recognizing GDCH as a vasculitis mimicker, ensuring that clinicians carefully evaluate patients presenting with gastrointestinal symptoms suggestive of vasculitis. A multidisciplinary approach involving rheumatologists, gastroenterologists, and radiologists is essential for accurate diagnosis and optimal management.

## Conclusions

Our case underscores the importance of considering alternative diagnoses in the clinical reasoning of vasculitis. The exclusion of vasculitis mimickers is a critical step when evaluating patients with suspected vasculitis. In the context of gastrointestinal vasculitis symptoms, vascular malformations should be actively investigated as differential diagnoses. When vasculitis is strongly suspected in life-threatening scenarios, prompt therapeutic intervention may be necessary before a definitive diagnosis is established. However, achieving an accurate diagnosis is essential for optimizing management and preventing unnecessary morbidity associated with both vasculitis and GDCH.
